# Global burden of esophageal cancer attributable to smoking: a systematic analysis for the Global Burden of Disease Study 2019

**DOI:** 10.3389/fonc.2023.1223164

**Published:** 2023-08-09

**Authors:** Shilong Wu, Wenfa Jiang, Jiufei Li, Zeqin Wu, Chenyang Xu, Ning Xie

**Affiliations:** Department of Thoracic Surgery, Ganzhou People’s Hospital, Ganzhou, China

**Keywords:** mortality, disability-adjusted life-years, esophageal cancer, smoking, global burden disease

## Abstract

**Background:**

Epidemiological trends of esophageal cancer attributable to smoking remain unclear. This study aimed to estimate the spatiotemporal trends of the esophageal cancer burden attributable to smoking to assist in global esophageal cancer prevention and smoking cessation.

**Methods:**

Data on esophageal cancer attributable to smoking were obtained from the Global Burden of Disease Study 2019. The number and age-standardized rates of esophageal cancer mortality (ASMR) and disability-adjusted life years (ASDR) were analyzed by age, sex, and location. Joinpoint regression analysis was used to analyze the temporal trends of esophageal cancer burden attributable to smoking over 30 years.

**Results:**

In 2019, the number of global esophageal cancer deaths and disability-adjusted life years (DALYs) attributable to smoking was approximately 203,000 and 475 million, respectively. The global esophageal cancer deaths and DALYs due to smoking were approximately 1.5-fold increased from 1990 to 2019, but the corresponding ASMR and ASDR had decreased. The heaviest burden occurred in East Asia, Mongolia, and the middle socio-demographic index (SDI) region. The male-to-female ratio was approximately 12.7 in the esophageal cancer deaths and DALYs and was approximately 14.3 in the ASMR and ASDR. The heaviest burden appeared in the 60–74 years age group. The estimated annual percentage change (EAPC) in ASMR was highly negatively associated with ASMR in 1990 (ρ = −0.41, p < 0.001) and SDI in 2019 (ρ = −0.29, p < 0.001).

**Conclusion:**

Despite reductions in ASMR and ASDR, the esophageal cancer burden attributable to smoking remains heavy, especially in middle SDI regions. Active tobacco control can reduce esophageal cancer burden.

## Introduction

Cancer ranks as a leading cause of premature death and an increased burden for the health system (1). According to the estimates from the GLOBOCAN 2020 database, there were approximately 604,000 new cases of esophageal cancer globally and approximately 544,000 deaths. Esophageal cancer is the seventh most common cancer and the sixth most common cause of cancer mortality worldwide (2). The major histological subtypes of esophageal cancer are esophageal squamous cell carcinoma (ESCC) and esophageal adenocarcinoma. Esophageal adenocarcinoma is linked to obesity, smoking, and gastroesophageal reflux disease. Over 85% of esophageal cancer cases are ESCC, and ESCC is linked to alcohol and tobacco consumption (3). The epidemiology and etiology of esophageal cancer may vary based on ethnicity or region (4).

With more than 1 billion people smoking tobacco regularly in 2019, almost 8 million deaths and 200 million disability-adjusted life years (DALYs) were attributable to smoking (5). Tobacco smoke contains more than 4,000 compounds, more than 60 of which are known carcinogens (6). In addition to the direct impacts of carcinogenic compounds, heightened inflammation due to tobacco smoking plays a role in increasing the risk of cancers (7). At the global level, 39.0% of esophageal cancer DALYs is attributable to tobacco smoking (8). Both the main histological subtypes of esophageal cancer are linked to smoking (8). Studies have shown that, compared with non-smokers, current smokers have a three- to sevenfold higher risk of developing ESCC, and the risk of ESCC is higher than the risk of esophageal adenocarcinoma (9). Moreover, smoking has an adverse effect on survival after esophageal cancer diagnosis (10).

The epidemiological features of esophageal cancer burden attributable to smoking at the global, national, and regional levels are unclear. In this analysis of data from the Global Burden of Diseases, Injuries, and Risk Factors Study (GBD) 2019, we estimate the esophageal cancer burden and the spatiotemporal trend attributable to smoking, assisting in esophageal cancer prevention and tobacco control.

## Materials and methods

### Study data

Data on the global burden of esophageal cancer attributable to smoking were obtained from GBD 2019 (http://ghdx.healthdata.org/gbd-results-tool), which include 204 countries and territories that are classified into 21 regions in terms of geography and into five groups (low, low-middle, middle, high-middle, and high) in terms of the socio-demographic index (SDI). The SDI is a composite indicator of development status related to health outcomes. The SDI ranges from 0 (worst) to 1 (best) and is composed of lag distributed income per capita, the total fertility rate under 25 years old, and mean education for those aged 15 years and over (4). SDI data were obtained from World Bank. The number of esophageal cancer deaths, DALYs, age-standardized mortality rate (ASMR), and age-standardized DALY rate (ASDR) attributable to smoking from 1990 to 2019 were extracted.

### Statistical analysis

Data of deaths, DALYs, ASMR, and ASDR were presented as numbers with 95% uncertainty intervals (UIs). The attributable burdens were analyzed by age, sex, and location. The estimated annual percentage change (EAPC) and its 95% confidence interval (CI) was calculated to measure the trends of ASMR and ASDR. The details of the calculation method were described by Xiaorong et al. (11). The ASMR or ASDR was considered to be in an increasing trend if the EAPC estimation and its lower boundary of 95% CI were both >0 and to be a decreasing trend if the EAPC estimation and its upper boundary of 95% CI were both <0. Otherwise, the ASMR or ASDR was considered to be stable over time. All the above-mentioned analyses were performed with the R program (R Core Team, version 3.6.2, Vienna, Austria).

The Joinpoint regression model was used to calculate the annual percentage change and the corresponding 95% CI to evaluate the temporal trends in ASMR and ASDR. A piecewise linear regression method was used to evaluate the trends by connecting several line segments on a logarithmic scale at the points (12). The Joinpoint software (version 4.9.1.0) was used to perform the Joinpoint regression analysis. Annual percentage change (APC) was calculated, and a p-value of less than 0.05 was considered statistically significant.

## Results

### Global burden of esophageal cancer attributable to smoking in 2019

Globally, the number of esophageal cancer deaths and DALYs attributable to smoking was approximately 203,330 and 4,746,520 in 2019, among which the male-to-female ratio was approximately 12.7 esophageal cancer deaths and DALYs. The esophageal cancer ASMR and ASDR due to smoking were approximately 2.48 and 56.71 per 100,000 population, among which the male-to-female ratio was approximately 14.3 in esophageal cancer ASMR and ASDR. Between 1990 and 2019, global ASMR decreased by 27.49%, and ASDR decreased by 32.79%. The esophageal cancer deaths and DALYs due to smoking were approximately 1.5-fold increased from 1990 to 2019, but the corresponding ASMR and ASDR had decreased, among which EAPC in ASMR and ASDR in men was more than those in women (**Table 1**).

**Table 1:** Global burden of esophageal cancer attributable to smoking in 1990 and 2019 and the temporal trends from 1990 to 2019.

Characteristics	1990				2019				EAPC (1990–2019)	
	Deaths cases	ASMR per 100,000	DALYs	ASDR per 100,000	Deaths cases	ASMR per 100,000	DALYs	ASDR per 100,000	ASMR	ASDR
	No. × 10^3^ (95% UI)	No. (95% UI)	No. × 10^3^ (95% UI)	No. (95% UI)	No. × 10^3^ (95% UI)	No. (95% UI)	No. × 10^3^ (95% UI)	No. (95% UI)	No. (95% CI)	No. (95% CI)
Overall	134.68 (105.31–152.93)	3.42 (2.69–3.86)	3,475.3 (2,674.42–3,967.05)	84.38 (65.34–96.3)	203.33 (170.46–236.51)	2.48 (2.08–2.89)	4,746.52 (3,983.52–5,544.23)	56.71 (47.57–66.14)	−1.32 (−1.59 to −1.04)	−1.57 (−1.86 to −1.28)
Sex										
Male	120.29 (92.3–137.58)	6.7 (5.22–7.62)	3,160.59 (2,404.54–3,635.36)	162.14 (123.89–186.04)	187.23 (156.03–219.35)	4.95 (4.12–5.78)	4,425.43 (3,688.96–5,188.28)	111.18 (92.75–130.29)	−1.23 (−1.49 to −0.97)	−1.48 (−1.76 to −1.2)
Female	14.4 (11.14–17.35)	0.69 (0.53–0.83)	314.71 (242.66–382.3)	14.73 (11.41–17.87)	16.09 (12.86–19.29)	0.37 (0.29–0.44)	321.09 (260.16–383.91)	7.33 (5.94–8.76)	−2.6 (−2.99 to −2.22)	−2.81 (−3.16 to −2.46)
SDI region										
High-middle SDI	39.34 (32.46–44.94)	3.66 (3.02–4.18)	1,018.86 (838.19–1,167.59)	92.06 (75.94–105.47)	62.58 (47.38–75.74)	3.03 (2.3–3.67)	1,457.73 (1,118.09–1,772.47)	70.49 (54.07–85.52)	−0.9 (−1.15 to −0.64)	−1.16 (−1.43 to −0.88)
High SDI	24.7 (22.59–26.69)	2.38 (2.18–2.57)	587.8 (541.27–632.07)	58.43 (53.84–62.79)	33.4 (29.39–37.68)	1.78 (1.58–2)	717.17 (639.68–797.82)	41.47 (37.1–46.1)	−1.13 (−1.24 to −1.01)	−1.32 (−1.43 to −1.21)
Low-middle SDI	10.95 (9.21–14.89)	1.88 (1.6–2.57)	298.98 (248.54–400.47)	46.39 (38.81–62.74)	18.77 (15.34–29.17)	1.4 (1.15–2.2)	481.96 (391.38–739.37)	33.72 (27.46–51.71)	−1.12 (−1.22 to −1.03)	−1.18 (−1.27 to −1.09)
Low SDI	3.47 (2.64–4.32)	1.51 (1.16–1.87)	95.61 (71.72–119.74)	37.35 (28.24–46.55)	5.95 (4.45–7.79)	1.18 (0.89–1.53)	163.43 (118.93–216.68)	29.33 (21.73–38.52)	−0.93 (−1 to −0.85)	−0.91 (−0.99 to −0.82)
Middle SDI	56.19 (33.02–67.5)	5.61 (3.36–6.72)	1,473.31 (858.89–1,795.34)	135.13 (79.41–162.86)	82.58 (64.27–100.31)	3.41 (2.64–4.14)	1,924.97 (1,514.17–2,332.74)	74.66 (58.57–90.4)	−1.96 (−2.36 to −1.56)	−2.28 (−2.69 to −1.87)
GBD region										
Andean Latin America	0.09 (0.07–0.11)	0.45 (0.34–0.54)	2.08 (1.58–2.56)	10.08 (7.7–12.37)	0.15 (0.11–0.2)	0.27 (0.19–0.37)	3.24 (2.3–4.51)	5.78 (4.12–8.01)	−1.64 (−1.75 to −1.53)	−1.81 (−1.94 to −1.68)
Australasia	0.42 (0.37–0.47)	1.79 (1.57–1.99)	9.7 (8.58–10.78)	41.57 (36.91–46.18)	0.5 (0.41–0.6)	1.02 (0.85–1.22)	10.93 (9.17–12.85)	24.1 (20.2–28.17)	−2.03 (−2.09 to −1.97)	−1.94 (−1.99 to −1.89)
Caribbean	0.36 (0.32–0.41)	1.42 (1.23–1.59)	8.66 (7.54–9.83)	33.1 (28.85–37.61)	0.68 (0.55–0.82)	1.3 (1.05–1.57)	16.92 (13.58–20.62)	32.29 (25.91–39.24)	0 (−0.15 to 0.15)	0.25 (0.1–0.4)
Central Asia	2.06 (1.86–2.26)	4.37 (3.93–4.81)	55.71 (50.04–61.27)	112.79 (101.31–124.14)	1.55 (1.32–1.95)	2.16 (1.83–2.68)	41.37 (34.68–52.74)	51.73 (43.78–65.53)	−2.54 (−2.79 to −2.29)	−2.85 (−3.11 to −2.6)
Central Europe	2.16 (1.96–2.34)	1.45 (1.32–1.57)	59.82 (54.5–64.69)	40.2 (36.67–43.48)	2.67 (2.22–3.15)	1.31 (1.08–1.54)	68.15 (56.26–81.2)	35.35 (29.05–42.1)	−0.5 (−0.6 to −0.4)	−0.64 (−0.76 to −0.52)
Central Latin America	0.66 (0.57–0.75)	0.84 (0.72–0.95)	16.18 (13.77–18.44)	18.99 (16.25–21.48)	0.92 (0.71–1.16)	0.4 (0.31–0.5)	21.46 (16.34–27.47)	8.97 (6.86–11.45)	−2.75 (−2.85 to −2.64)	−2.72 (−2.83 to −2.61)
Central Sub-Saharan Africa	0.51 (0.23–0.72)	2.22 (1.01–3.14)	14.67 (6.47–20.51)	57.27 (25.65–79.97)	0.8 (0.42–1.16)	1.47 (0.79–2.14)	23.07 (12.08–33.79)	38.16 (20.37–55.8)	−1.76 (−1.98 to −1.54)	−1.74 (−1.95 to −1.53)
East Asia	76.97 (49.28–92.68)	9.02 (5.88–10.79)	1,987.66 (1,251.63–2,422.13)	213.99 (136.15–259.34)	125.7 (95.84–155.77)	6.06 (4.63–7.49)	2,875.59 (2,203.98–3,577.91)	132.28 (101.86–164)	−1.61 (−2.01 to −1.21)	−1.89 (−2.32 to −1.47)
Eastern Europe	5.35 (4.77–5.92)	1.85 (1.65–2.05)	152.11 (135.54–169.35)	52.8 (47.07–58.67)	5.06 (4.23–5.97)	1.47 (1.23–1.74)	137.93 (115.22–163.25)	41.37 (34.59–48.99)	−1.18 (−1.39 to −0.97)	−1.27 (−1.51 to −1.04)
Eastern Sub-Saharan Africa	1.9 (1.26–2.55)	2.62 (1.75–3.5)	51.93 (33.61–70.31)	64.66 (42.34–87.2)	3.3 (2.24–4.73)	2.08 (1.44–2.95)	90.95 (60.8–132.64)	51.75 (34.93–74.65)	−0.91 (−1.05 to −0.78)	−0.9 (−1.05 to −0.75)
High-income Asia Pacific	5.66 (5.19–6.1)	2.8 (2.56–3.01)	138.16 (125.92–149.11)	66.28 (60.52–71.46)	6.95 (6–7.93)	1.54 (1.34–1.75)	135.32 (119.01–154.44)	33.76 (29.63–38.49)	−2.28 (−2.45 to −2.11)	−2.61 (−2.81 to −2.41)
High-income North America	6.84 (6.04–7.6)	1.98 (1.76–2.19)	161 (145.11–175.91)	48.82 (44.13–53.11)	10.82 (9.3–12.37)	1.71 (1.48–1.94)	235.49 (206.91–263.96)	39.01 (34.49–43.52)	−0.61 (−0.73 to −0.48)	−0.88 (−1 to −0.76)
North Africa and the Middle East	1.35 (0.98–1.63)	0.81 (0.6–0.97)	36.92 (25.6–45.03)	19.93 (14.23–24.2)	3.13 (2.39–3.7)	0.76 (0.59–0.89)	81.06 (59.5–96.66)	17.51 (13.17–20.67)	−0.21 (−0.25 to −0.16)	−0.47 (−0.53 to −0.41)
Oceania	0.02 (0.02–0.03)	0.8 (0.56–1.17)	0.69 (0.47–0.99)	20.6 (14.18–29.65)	0.05 (0.03–0.07)	0.69 (0.48–0.97)	1.41 (0.95–2)	17.56 (12.15–24.83)	−0.52 (−0.61 to −0.43)	−0.54 (−0.64 to −0.44)
South Asia	8.1 (6.63–10.11)	1.5 (1.25–1.86)	224.65 (182.21–284.52)	36.46 (29.76–45.49)	13.27 (10.24–18.92)	0.96 (0.75–1.38)	347.99 (267.73–492.69)	23.53 (18.19–33.26)	−1.67 (−1.77 to −1.56)	−1.62 (−1.71 to −1.53)
Southeast Asia	2.82 (2.31–3.33)	1.13 (0.94–1.34)	76.71 (61.27–90.93)	27.92 (22.6–33.17)	5.81 (4.69–7.13)	0.96 (0.78–1.17)	154.63 (122.82–188.9)	23.4 (18.66–28.61)	−0.66 (−0.72 to −0.6)	−0.68 (−0.73 to −0.62)
Southern Latin America	1.39 (1.22–1.56)	2.99 (2.63–3.36)	34.58 (30.69–38.4)	73.56 (65.33–81.72)	1.31 (1.11–1.53)	1.56 (1.33–1.83)	30.06 (25.67–34.48)	36.79 (31.39–42.24)	−2.52 (−2.67 to −2.36)	−2.69 (−2.84 to −2.54)
Southern Sub-Saharan Africa	1.53 (1.12–1.97)	5.59 (4.06–7.21)	43.47 (31.57–56.22)	146.76 (106.51–189.45)	1.78 (1.45–2.22)	3.15 (2.58–3.86)	49.03 (39.4–62.24)	80.71 (65.22–101.44)	−2.83 (−3.43 to −2.23)	−2.94 (−3.54 to −2.33)
Tropical Latin America	3.16 (2.83–3.48)	3.52 (3.16–3.89)	85.41 (76.72–93.83)	87.52 (78.6–96.23)	3.86 (3.29–4.46)	1.58 (1.35–1.82)	98.3 (83.47–113.73)	39.2 (33.35–45.38)	−2.66 (−2.83 to −2.49)	−2.66 (−2.86 to −2.46)
Western Europe	12.98 (11.83–14.19)	2.29 (2.1–2.5)	305.91 (279.09–331.95)	57.03 (51.93–61.74)	14.18 (12.48–16.16)	1.61 (1.42–1.81)	299.29 (262.81–336.26)	37.78 (33.12–42.22)	−1.34 (−1.41 to −1.26)	−1.54 (−1.62 to −1.47)
Western Sub-Saharan Africa	0.33 (0.25–0.42)	0.37 (0.28–0.47)	9.28 (6.9–12.03)	9.74 (7.31–12.57)	0.85 (0.56–1.12)	0.45 (0.3–0.58)	24.31 (15.88–32.42)	11.62 (7.63–15.28)	0.97 (0.8–1.13)	0.91 (0.74–1.08)

DALYs, disability-adjusted life years; ASDR, age-standardized DALY rate; No., number; UI, uncertainty interval; ASMR, age-standardized mortality rate; EAPC, estimated annual percentage change; CI, confidence interval; SDI, sociodemographic index.

From among 21 GBD regions, the heaviest burden was in East Asia in 2019, with over 60% of deaths (125.7 per 1,000 population) and DALYs (2,875.59 per 1,000 population). In 2019, the highest regional ASMR (6.06 per 100,000 population in East Asia) was 22.4 times higher than the lowest (0.27 per 100,000 population in Andean Latin America). The highest regional ASDR (132.28 per 100,000 population in East Asia) was 22.9 times higher than the lowest (5.78 per 100,000 population in Andean Latin America). The ASMR and ASDR decreased in most GBD regions except in the Caribbean and Western Sub-Saharan Africa. In the meantime, Southern Sub-Saharan Africa had the fastest decrease in ASMR and ASDR, with EAPC of −2.83 in ASMR and EAPC of −2.94 in ASDR (**Table 1**).

**Figure 1:**
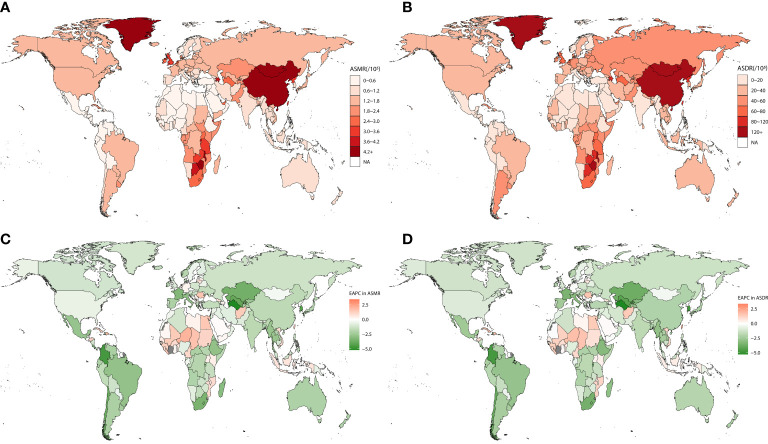
The spatial distribution of esophageal cancer ASMR **(A)** and ASDR **(B)** attributable to smoking in 2019 and the EAPC in esophageal cancer ASMR **(C)** and ASDR **(D)** attributable to smoking. ASMR, age-standardized mortality rate; ASDR, age-standardized disability-adjusted life-year rate; EAPC, estimated annual percentage change.

At the country level, China had the largest number of esophageal cancer deaths (123,070 [95% UI 93,222–153,229]) and DALYs (2,801,241 [95% UI 2,133,864–3,501,723]) attributable to smoking in 2019. Mongolia ranked first in the number of esophageal cancer ASMR (7.56 per 100,000 population) (**Figure 1A**) and ASDR (164.97 per 100,000 population) (**Figure 1B**) attributable to smoking in 2019. Mongolia and Greenland were the top two in ASMR and ASDR in 2019. The fastest decrease in ASMR (−5.21, 95% CI: −4.46 to −5.95) (**Figure 1C**) and ASDR (−5.31, 95% CI: −4.56 to −6.05) (**Figure 1D**) occurred in Turkmenistan, and the fastest increase in ASMR (3.40, 95% CI: 3.18 to 3.61) and ASDR (3.35, 95% CI: 3.13 to 3.57) occurred in São Tomé and Principe. The ASMR and ASDR had decreased in most countries, and most of the growth was in Africa.

At the SDI region level, the middle SDI region had the most smoking-related esophageal cancer deaths (82.58 per 1,000 population) and DALYs (1,924.97 per 1,000 population) in 2019. All SDI region groups had a decrease in ASMR and ASDR from 1990 to 2019. The middle SDI region had the fastest decrease in ASMR and ASDR.

### Global burden of esophageal cancer attributable to smoking by age and sex

**Figure 2:**
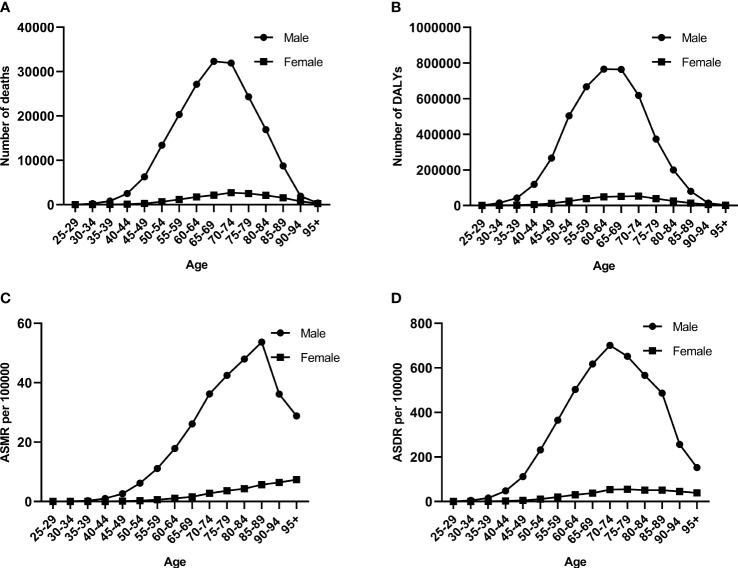
The burden of esophageal cancer attributable to smoking among different genders and ages in 2019. **(A)** Number of deaths. **(B)** Number of DALYs. **(C)** ASMR rates. **(D)** ASDR rates. DALYs, disability-adjusted life years; ASMR, age-standardized mortality rate; ASDR, age-standardized DALY rate.

In 2019, the number of esophageal cancer deaths attributable to smoking first increased and then decreased with age similarly in men and women, with the peak point appearing in 65–69 and 70–74 years old (**Figure 2A**). More esophageal deaths occurred in men compared to women, and the ASMR in men was correspondingly higher than that in women. The ASMR rapidly increased before 85–89 years old and rapidly decreased in men but steadily increased in women (**Figure 2C**).

The number of esophageal cancer DALYs showed a similar pattern to that of deaths in men and women, but the peak point appeared at 60–64 years old (**Figure 2B**). The number of esophageal cancer DALYs and ASDR was more in men than in women. The trend of the ASDR was similar to that of the number of esophageal cancer DALYs in men, but the peak point of ASDR was 10 years advanced compared to that of the number of DALYs (**Figure 2D**). The ASDR in women gradually increased before 70–74 years old and then remained stable.

### Factors associated with esophageal cancer burden attributable to smoking

**Figure 3:**
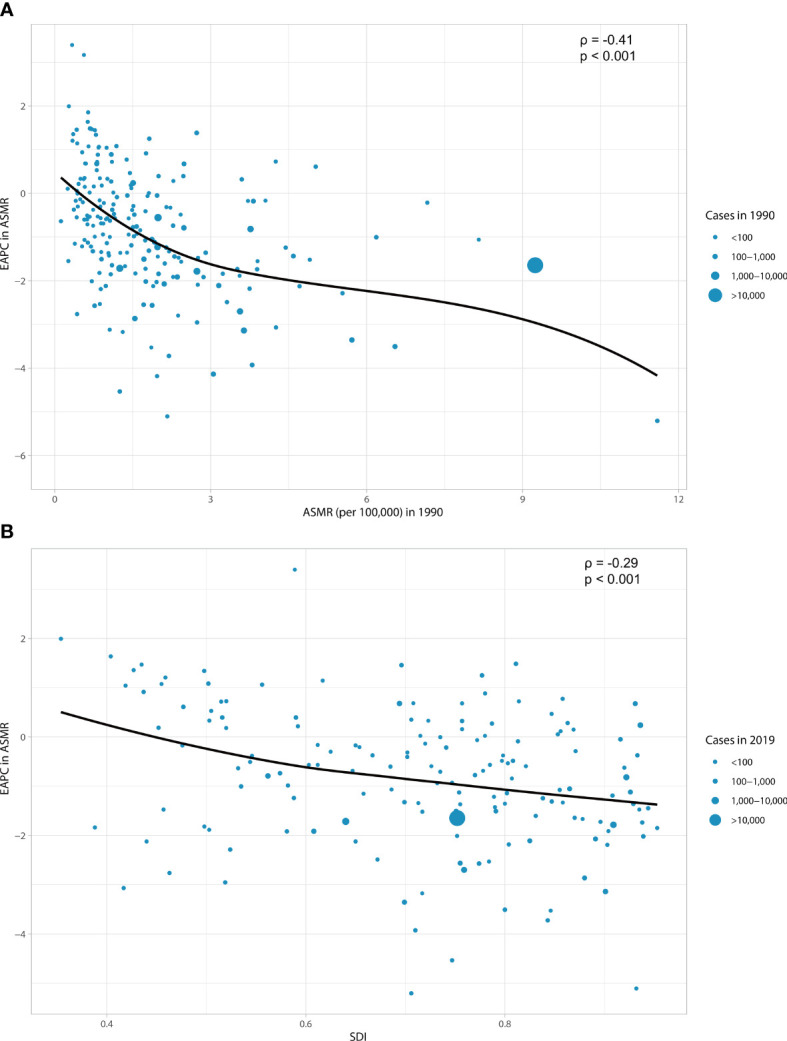
The correlation between EAPC in ASMR and ASMR in 1990 **(A)** and between EAPC in ASMR and SDI in 2019 **(B)**. The circles represent countries that were extracted from SDI data. The size of circles represents the cases of esophageal cancer attributable to smoking. EAPC, estimated annual percentage change; ASMR, age-standardized mortality rate; SDI, socio-demographic index.

As shown in **Figure 3**, a significant association was detected between EAPC and ASMR, and SDI. The ASMR of esophageal cancer in 1990 reflects the disease reservoir at baseline (**Figure 3A**). A significant negative association was found between EAPC and ASMR in 1990 (ρ = −0.41, p < 0.001). The SDI in 2019 can serve as a substitute for evaluating developmental conditions and health care (**Figure 3B**). The relationship between EAPC and SDI was significantly negative (ρ = −0.29, p < 0.001). The countries with higher SDI showed lower burdens.

**Figure 4:**
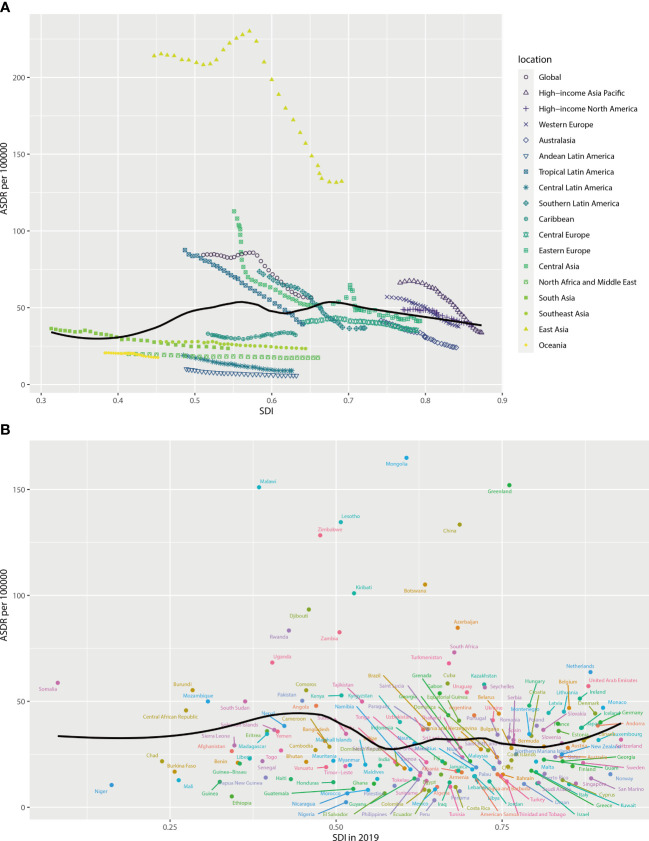
ASDR for esophageal cancer attributable to smoking for regions **(A)** and countries **(B)** by SDI. ASDR, age-standardized disability-adjusted life-year rate; SDI, socio-demographic index.

As shown in **Figure 4**, the estimated regional and national ASDRs in relation to SDI are compared with the expected level for each location based on SDI. Most of the high SDI regions such as high-income North America, Western Europe, Eastern Europe, and Australasia closely followed expected trends over the period. The observed patterns of middle SDI regions had a large variation. Among these middle SDI regions, some stayed below expected levels with minor changes in ASDR over the period, and some others stayed above expected levels with decreasing or fluctuating ASDR (**Figure 4A**). In 2019, there was a positive association between the expected ASDR and SDI at the national level when the SDI was less than 0.4 or greater than 0.8 and a negative association when the SDI was in the middle group (**Figure 4B**).

### Temporal trends of esophageal cancer burden attributable to smoking

**Figure 5:**
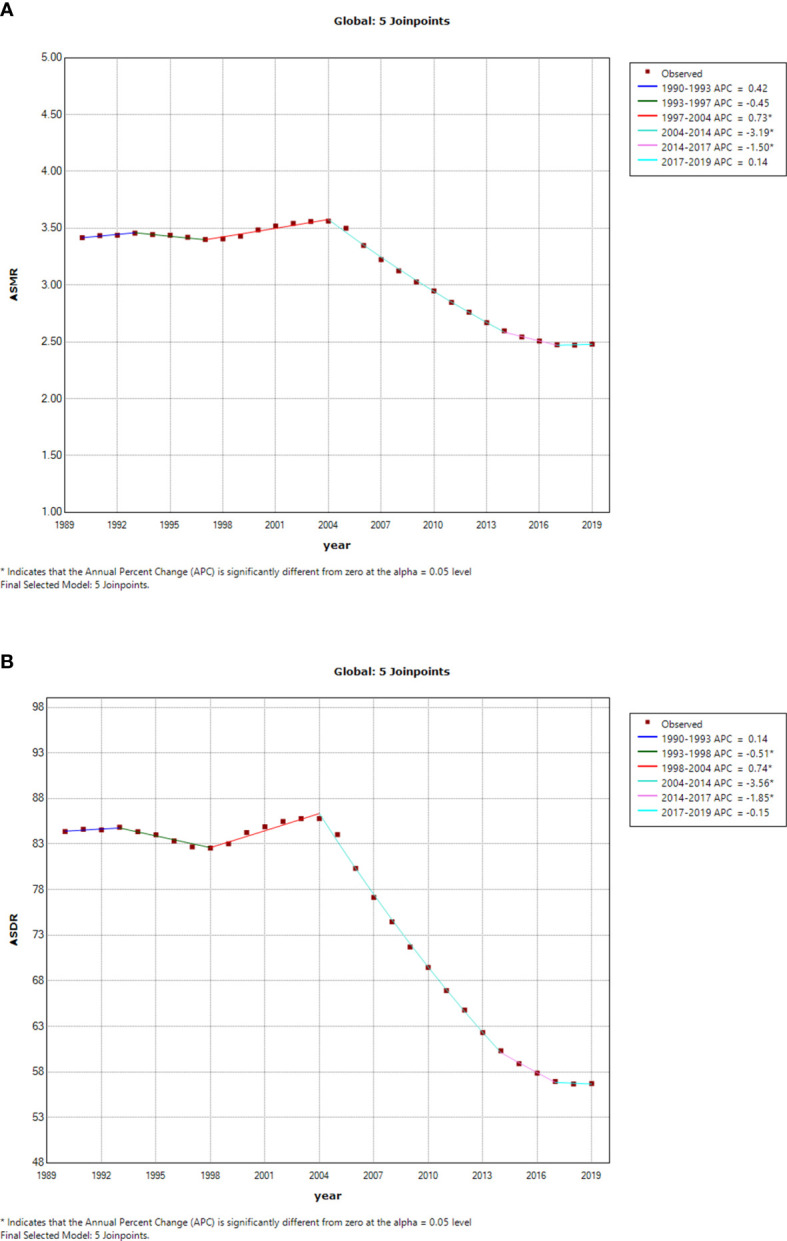
Temporal trends of global esophageal cancer burden attributable to smoking from 1990 to 2019. **(A)** ASMR of esophageal cancer attributable to smoking. **(B)** ASDR of esophageal cancer attributable to smoking. ASMR, age-standardized mortality rate; ASDR, age-standardized disability-adjusted life-year rate.

The esophageal cancer ASMR and ASDR due to smoking increased steadily from 1990 to 2004 and then experienced a significant decrease until 2019 (**Figure 5**). The esophageal cancer ASMR decreased with different APCs since 2004, and the most significant decrease occurred between 2004 and 2014 (APC = −3.19%, p < 0.05) (**Figure 5A**). Similarly, the esophageal cancer ASDR obviously fell from 2004 to 2019 (2004–2014: APC = −3.56%, p < 0.05; 2014–2017: APC = −1.85%, p < 0.05) (**Figure 5B**).

## Discussion

Despite decreases in ASMR and ASDR, esophageal cancer remains a major cause of cancer burden around the world. In addition to tobacco smoking, tobacco chewing, alcohol consumption, obesity, and low intake of fruit were also demonstrated to account for most of the burden (8). In this analysis, we found that the absolute burden of esophageal cancer attributable to smoking had been decreasing globally though the number of deaths and DALYs increased since 1990. The burden of esophageal cancer attributable to smoking posed enormous challenges to men, the elderly, middle SDI regions, and East Asia. The spatiotemporal distribution of esophageal cancer attributable to smoking was heterogeneous, which showed a complicated connection between geographical variations and economic development.

Although the trend of esophageal cancer in ASMR and ASDR attributable to smoking has decreased at the global level from 1990 to 2019, the corresponding absolute numbers of esophageal cancer deaths and DALYs have increased as a result of population explosion and aging. The trends indicate that esophageal cancer remains a major cause of cancer mortality and burden across the world. On the one hand, aging is a risk for cancer development, and the morbidity and mortality rates of esophageal cancer start climbing at age 50 years (8, 13). The World Health Organization estimated that the global population over 60 years was expected to double by 2050. On the other hand, many effective tobacco control programs and policies have greatly reduced the prevalence of tobacco use in recent decades, including tobacco taxes, smoking bans in public places, and the WHO Framework Convention on Tobacco Control (14, 15). Although the global smoking prevalence has decreased, the absolute number of smokers is still increasing because of population growth. This phenomenon follows a similar finding of the increasing number of esophageal cancer deaths and DALYs attributable to smoking but decreasing prevalence rates globally. The temporal variation of ASMR and ASDR in esophageal cancer attributable to smoking is extensive and has decreased rapidly from 2004 to 2014.

For different genders, our results showed that the esophageal cancer burdens attributable to smoking were more remarkable for men, among which men account for approximately 92% in 2019. Moreover, the smoking prevalence in men is higher than in women (16). The study led by GBD 2019 Tobacco Collaborators showed that men accounted for four-fifths of the total cancer deaths and DALYs due to tobacco smoking in 2019 (5). In 2017, the ASMR of esophageal cancer was 2.7 times higher, mortality was 2.9 times higher, and DALYs were 3.0 times higher in men than in women (8). The results show that the esophageal cancer burdens are greater for men. Globally, the number of deaths due to cancer increased with age and peaked at 65–74 years in 2019. The harm of smoking is accumulated over time (17). Our study also showed similar results that the number of esophageal cancer deaths attributable to smoking peaked at 65–74 years in 2019. Some studies have shown that most smokers start smoking at younger ages, and a person is less likely to become a smoker after the age of 25 years (18–20). It is an effective way to reduce the smoking-induced burden that targets these young people by implementing and enforcing evidence-based tobacco control policies (20).

The esophageal cancer burden attributable to smoking varied substantially across regions and nations. The ASMR and ASDR in the highest-burden GBD regions are 23 times higher than those in the lowest regions. The ASMR and ASDR of esophageal cancer attributable to smoking in most of the regions displayed a decreasing trend, but an increasing trend occurred in Western Sub-Saharan Africa. The study led by Jiahui Fan et al. found that a lower estimated supply of Mg, SE, Fe, and Zn in diets may be related to the rising trend of this region (4). Otherwise, it could be due to improvements in cancer registry systems in Western Sub-Saharan Africa (8). Our results show that the amplitude in ASMR variations, namely, EAPC in ASMR, between 1990 and 2019 was significantly negatively associated with baseline ASMR and SDI in 2019. The ASMR and ASDR in five SDI regions showed great variation, with the highest ASMR and ASDR in the middle SDI regions, but the middle SDI regions showed the most significant downward trend since 1990. For the low SDI regions, the esophageal cancer burden attributable to smoking was the lowest and showed slowly decreasing trends. The esophageal cancer burden was higher in middle and high-middle SDI regions (8). SDI was a positive association with the burden of cancer attributable to smoking, probably because smoking was more prevalent among higher SDI regions (21, 22). Strengthening tobacco control in higher SDI could have tremendous effects on the esophageal cancer burden attributable to smoking.

As far as we know, this study is the first comprehensive study that used GBD data to analyze the spatiotemporal trends of esophageal cancer burden attributable to smoking. Our study provides a novel insight to mitigate esophageal cancer burden by reducing the modifiable risk factors of the disease. However, some limitations of this study should be noted. First, some countries and regions did not have complete mortality or DALY data, especially in lower SDI countries and regions. Second, the accuracy of data for esophageal cancer mortality attributable to smoking depends on disease monitoring systems or cancer registries. Some regions lack disease monitoring systems or cancer registries. Our estimates of esophageal cancer burden attributable to smoking were restricted by the available data.

## Conclusions

In conclusion, despite increases in the number of deaths and DALYs, great efforts have been made on tobacco control, and the global burden of esophageal cancer attributable to smoking continues to reduce. Smoking should be regarded as an important and preventable risk factor for the burden of esophageal cancer, especially in the elderly, men, and people in middle SDI regions. Our study provides evidence regarding the heavy esophageal cancer burden resulting from smoking and assists in implementing effective measures to lessen the esophageal cancer burden.

## Data availability statement

The original contributions presented in the study are included in the article/supplementary material. Further inquiries can be directed to the corresponding author.

## Author contributions

Conception and design: SW, WJ, and NX. Administrative support: NX and CX. Collection and assembly of data: SW. Data analysis and interpretation: SW, JL, and ZW. Manuscript writing: all authors. Final approval of manuscript: all authors. All authors contributed to the article and approved the submitted version.
